# Evaluation of the Relationship between the Levels of Patience and Tranquillity and Conflict Resolution Styles of Executive Nurses

**DOI:** 10.1155/2024/6651729

**Published:** 2024-03-13

**Authors:** Ayse Gumusler Basaran, Bahar Kefeli Col, Burcu Genc Kose

**Affiliations:** ^1^Faculty of Health Science, Recep Tayyip Erdogan University, Rize, Türkiye; ^2^Guneysu Vocational School of Physical Therapy and Rehabilitation, Recep Tayyip Erdogan University, Rize, Türkiye; ^3^Vocational School of Health Services, Recep Tayyip Erdogan University, Rize, Türkiye

## Abstract

**Aims:**

This study examined nurse managers' conflict resolution styles, tranquillity and patience levels, and their relationships.

**Background:**

Managers are supposed to know how to manage conflict to reduce the destructive effects of conflict and create constructive effects.

**Methods:**

The study was a descriptive cross-sectional study and in a city centre in Karadeniz region, in May 2022. It was aimed to reach all executive nurses but was completed with 41 executive nurses. The data were collected face to face using a Sociodemographic Questionnaire, the Rahim Organizational Conflict Inventory, the Patience Scale, and the Tranquillity Scale.

**Results:**

51.2% experienced conflict with colleagues and 46.3% with other employees. In conflict management, the most commonly used style was integrating and the least was dominating, respectively. The Patience Scale score (39.15 ± 6.09) and Tranquillity Scale score (3.70 ± 0.70) were moderate. At the same time, long-term (10.19 ± 2.18), short-term (8.90 ± 2.54), and total patience scores were significantly lower in case of conflict with nurses. Interpersonal patience levels were significantly lower in case of conflict with other employees. There was a negative correlation between working as a manager and compromising style.

**Conclusion:**

It was concluded that executive nurses used the integrating style more, and their patience and tranquillity levels were moderate. In addition to using the integration style more, the fact that nurse managers have moderate levels of patience and calmness will reflect positively on the quality of patient care. It will also increase employee satisfaction. Increasing the level of peace in health institutions will support patience, happiness, and a sense of belonging among employees.

## 1. Introduction

Conflict is a disagreement or incompatibility between two or more people or groups, a clash of interest, power, and status [1] and occurs when conflicting activities happen [2]. Conflict is a contestation of interests, power, and status between two or more individuals or groups and occurs when conflicting activities occur. The differences in values, ideas, and attitudes, communication and coordination disorders, uncertainties about the management field, and sometimes the poor functioning of the management process can be considered the root causes of conflict in hospitals [1, 3, 4]. Conflict is not always positive or negative; it can signify danger or a harbinger of new opportunities. Whether conflict outcomes are positive or negative is related to how it is managed [1]. Constructively worked conflict is said to bring about healthy competition, strengthen team participation, and bridge the communication gap [5]. Therefore, conflict management is one of the skills managers require [6].

Conflict resolution is the method used to resolve conflicts in social situations [7]. Different strategies such as integration, obliging, dominating, compromising, and avoiding are used in conflict management. The “integration” strategy adds up to a high interest for self and others, plus involves preciseness, information exchange, and questioning. As for the strategy of “compromising,” the individual neglects his self-interest to satisfy the other party's anxiety, resulting in the situation's characterisation as obedience and compliance with the requests of the opposite party. In addition, it is used when the protection of relations with the other party is more important than the satisfaction of needs. Contrary to the strategy of “integration,” “domination” adds up to a high interest for oneself and a low interest for others. The dominant person will do anything to win, resulting in their ignorance of the other party's needs and expectations. Conflict is viable unless it is between the superior and the subordinate, but it can lead to a stalemate when both sides are on equal footing. The “avoiding” strategy involves low interest, both for oneself and others. In other words, an avoidant cannot satisfy both his anxiety and others. Instead of resolving the conflict, postponement can be used to gain time for one of the parties. The “obliging” strategy involves both parties giving up on something to reconcile mutually. Therefore, it is unsuitable for dealing with complex problems requiring a problem-solving approach. However, it is useful when building a consensus is impossible and both parties need a temporary solution to a complex issue [1, 8, 9].

Nurse managers experience conflict daily and are central to conflict management [10]. A study revealed that 61.4% of the nurses in charge of the service had conflicts with the nurses they worked with, most of which were caused by organisational reasons (61.5%) [11]. The leading causes of conflict between physicians and nurses are ambiguity in job descriptions, communication problems, rude and destructive behaviours of physicians, perception of the profession [12], and differences in doctors' demand for power and nurses' demand for influence [13]. In addition, other studies have found moderate to high levels of stress [14–16], job burnout [17], low job satisfaction [15, 18–21], and occupational fatigue [22] in nurses. Factors affecting stress and job satisfaction, such as high workload, shift work and overtime, staff shortage, job uncertainty, workplace violence, and managers' attitude [15, 16, 22–24] can also be considered as factors that cause conflict.

The reasons for the conflict among the executive nurses were affected by variables such as the hospital, age, and professional experience. In addition, the job description and workload caused the conflict [25]. Regarding conflict management, it was determined that the service managers used the integration strategy the most and avoidance the least [11]. Accordingly, nursing students mostly used the strategy of obliging and least domination [26]; in addition, the conflict strategies of university students did not differ according to gender [27]. Different strategies were used to resolve the conflict between doctors and nurses; the most used strategies were integration, compromising, and avoidance, whereas the least used one was dominating [12]. While determining the appropriate strategy in a conflict, considering its contribution to organisational effectiveness, satisfying social needs, and meeting members' ethical and moral needs are crucial. However, excessive integration and obliging strategies help solve strategic problems [1].

Patience, which is among the characteristics expected in managers [28, 29], is defined as a person's tendency to stand calmly in the face of disappointment, distress, or suffering [30]. The concept of patience gives the manager purpose, tolerance, openness to change, and empathy. It is expected by employees to be more compassionate, open-minded, and willing to manage any situation [29]. Patience has long been recognised as a human strength and a critical component of moral excellence [31]. Its structure saves the individual from negative emotions and increases life satisfaction [32]. The demonstration of true patience depends on both behavioural and emotional components [30]. Patience is positively related to subjective well-being, positive coping, and success [31]. It also includes powerful virtues, such as balance and justice. Patients have less negative affect, lower depression, fewer health problems, and increased life satisfaction [32]. Nurses' patience levels were above the average [33], and it was determined to be a component that affects nurses' resilience in Iran [34].

On the other hand, tranquillity reflects feelings such as comfort, calmness, and serenity, expressed by the characteristics of [35] individuals, such as harmony, balance, comfort, confidence, and inner peace [36]. When there is no threat in daily life, feelings of satisfaction, tranquillity, and well-being emerge, and people generally express their expectations from life in two different ways: tranquillity and happiness [35]. Although studies on patience and tranquillity in the healthcare field are limited, tranquillity and employee performance are positively associated [37]. In this respect, it is thought to be a pioneering study.

Conflicts can frequently emerge due to the complex structure of health services, which are also areas where different disciplines work together. They can happen between health personnel, resulting in medical errors with severe consequences if they are not resolved [38, 39]. Since all conflicts in health institutions will affect cooperation and the delivery of quality health services, managing the conflict with a positive outcome is crucial. Therefore, it is necessary to determine the use of conflict resolution strategies by executive nurses. In addition, nurses work in extraordinary situations such as pandemics. Also, these processes cause nurses to experience depression, stress, and anxiety [40]. These negative emotions can affect the level of patience and peace of mind of nurses. Patience and peace levels of nurse managers will be reflected on the working environment and nurses and will affect the quality of service. The level of patience and peace affected may also influence the choice of conflict resolution strategy. Therefore, this study aims to determine executive nurses' conflict resolution strategies, tranquillity, and patience levels and examine their relationship.

Research Questions.

1. Which conflict resolution strategies is the most used by nurse managers in conflict resolution?
2. Which levels of tranquillity and patience the nurse managers need?
3. Is there a relationship among conflict resolution strategies, tranquillity, and patience levels?

The hypotheses of this study are formulated as follows.

  Hypothesis 1. There is a relationship between the patience levels of nurse managers and their conflict resolution styles.
  Hypothesis 2. There is a relationship between the serenity levels of nurse managers and their conflict resolution styles.
  Hypothesis 3. Patience level and serenity level are related to each other.

## 2. Methods

### 2.1. Population and Sample of the Study

This study was conducted as a descriptive cross-sectional study. The study population comprised 60 executive nurses working in the Education and Research Hospital and the State Hospital in a city centre in Karadeniz region. It was aimed to reach all administrative nurses without dealing with the sample determination process. The study was completed with 41 executive nurses who agreed to participate and obtained consent in May 2022. 68% of the research population has been reached.

  Inclusion criteria were as follows:
  Working as an executive nurse
  Volunteering to participate in the study

### 2.2. Sampling Strategy

The questionnaires were distributed to the executive nurse and collected after they were filled out.

### 2.3. Data Collection Tools

The data were collected face to face using the Sociodemographic Questionnaire, the Rahim Organizational Conflict Inventory Scale, the Patience Scale, and the Tranquillity Scale.

#### 2.3.1. Sociodemographic Questionnaire

The questionnaire developed by the researcher consists of 11 questions, including age, gender, marital status, educational status, working time in the profession, working time in the current institution, working time as a manager, condition of a conflict with nurses, condition of a conflict with other employees, and state of finding oneself patient and peaceful.

#### 2.3.2. Rahim Organizational Conflict Inventory (ROCI-II)

This scale was developed by Rahim in 1983 to identify five different conflict management strategies. It was adapted into Turkish by Gumuseli and Taymaz and consisted of three forms. Form A includes conflict management strategies used by subordinates in conflicts with superiors. Unlike form A, form B holds strategies superiors use in conflicts with subordinates. Form C consists of strategies organisation members use in conflicts with their peers. In this study, form B, also called ROCI II, was used. The scale, which was designed in a 5-point Likert type, consists of 28 items and has options such as always (5), often (4), sometimes (3), rarely (2), and very rarely (1). It reveals individuals' use of five conflict management strategies to what extent. Also, it has the following five subdimensions: integration, compromising, dominating, avoiding, and obliging. Items “1, 5, 12, 22, 23, and 28” are related to the “integration” strategy, items “2, 11, 13, 19, and 24” to “compromising,” “8, 9, 18, 21, and 25” to “dominating,” “4, 7, 10, 14, 15, and 20” to “obliging,” and items “3, 6, 16, 17, 26, and 27” to the “avoiding” strategy. The score intervals of 4.20–5.00 (always), 3.40–4.19 (often), 2.60–3.39 (sometimes), 1.80–2.59 (rarely), and 1.00–1.79 (very infrequently) are used in grading and interpreting the weighted averages obtained from the scale. A high score from any subdimension indicates that that specific conflict management strategy is used more than others. In contrast, a low score from any subdimension shows that that particular strategy is used less than different strategies [8, 41]. Cronbach's *α* of the scale subscales was calculated as 0.81 [41]. In this study, Cronbach's *α* values of the scale were found in the range of 0.66–0.89.

#### 2.3.3. Patience Scale

The Patience Scale was developed by Schnitker, and its validation study in Turkish was conducted by Dogan and Gulmez. There are three subdimensions on the scale, namely, interpersonal patience: 1, 4, 7 (*r*), 9, 11; long-term patience (patience in life hardships) 2, 5, 8; and short-term patience (patience in daily life) 3, 6, 10 (*r*). It was designed in a 5-point Likert type with 11 items and covered the options between “strongly agree” and “strongly disagree.” Also, it contains nine positive and two opposing statements. Items 7 and 10 are reverse scored. The highest score on the scale is 55, and the lowest is 11 points. The highest and lowest score ranges that can be obtained from the subdimensions of the scale are interpersonal patience “5–25,” long-term patience “3–15,” and short-term patience “3–15.” A high score obtained from the scale indicates that the patience levels of the individuals are high, whereas a lower score shows a lower level of patience [30, 42]. Cronbach's *α* of the scale was found to be 0.78 [42]. The Cronbach's *α* value was found to be 0.68 in the study.

#### 2.3.4. Tranquillity Scale

The Tranquillity Scale developed by Demirci and Eksi is an 8-item one-dimensional scale. Items 5 and 6 are reverse scored. It was designed in a 5-point Likert type, including options such as not at all suitable for me (2), not suitable for me (3), somewhat suitable for me (4), fairly suitable for me (5), and completely suitable for me. The highest score on the scale is 40, and the lowest is 8 points. A high score obtained from the scale indicates that the tranquillity levels of the individuals are high, whereas a lower score shows a lower level of tranquillity. The Cronbach's *α* internal consistency coefficient of the scale was calculated as 0.78 [35]. Also, its Cronbach's *α* value was found to be 0.84 in the study.

### 2.4. Statistical Analysis

For the statistical analysis of the data, the SPSS Statistics 22 software was used. Elements such as percentage, mean, and standard deviation were used to analyse descriptive data. In addition, the Student t-test, Mann–Whitney *U* test, one-way ANOVA, Kruskal–Wallis test, pairwise comparisons, Tukey's HSD post hoc test, and the Pearson correlation analysis were used according to the normal distribution of quantitative data. In the correlation analysis, 0–0.39 was considered a weak correlation, 0.40–0.69 a moderate correlation, 0.70–0.89 a strong correlation, and 0.90–1.00 a powerful correlation [43]. The level of significance was accepted as *p* < 0.05.

### 2.5. Ethical Considerations

The study data were collected by obtaining the Ethics Committee's permission of the university numbered 2022/89 and institutional permissions from the relevant units. Participants agreed to participate voluntarily.

## 3. Results

**Figure 1:**
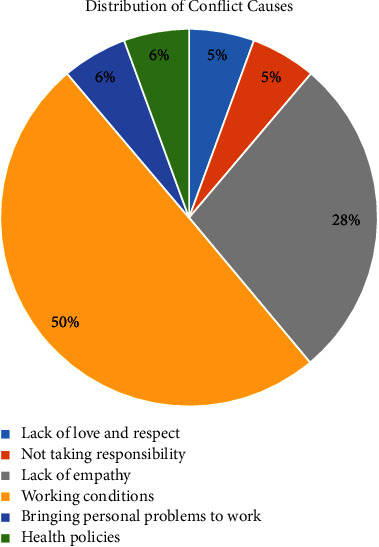
Reasons for conflict of executive nurses with colleagues.

37 (90.2%) of the executive nurses participating in the study were women, 38 (92.7%) were married, and their age average was 37.90 ± 7.35. If we look at their educational status, 27 (65.9%) of them have a bachelor's degree, 8 (19.5%) have an associate's degree, 4 (9.8%) are postgraduates, and 2 (4.9%) are high school graduates. Their working hours in the profession are 14.5 ± 7.31, in the current institution 11.8 ± 7.23, and as a manager 5.69 ± 5.07. 21 (51.2%) of the participants stated that they had conflicts with their colleagues, 19 (46.3%) had conflicts with other employees, 38 (92.7%) were patient, and 29 (70.7%) were peaceful. When the reasons stated by those who have conflicts with their colleagues are classified, we develop six themes. The highest cause of conflict is due to working conditions such as lack of personnel and heavy workload, as shown in Figure 1.

When we group the themes as per the literature, 8 (44.4%) of the conflicts are happening due to individual reasons (lack of love and respect, not taking responsibility, lack of empathy, and personal problems), 10 (55.6%) of them due to organisational reasons (working conditions and health policies).

Observations revealed that executive nurses always used the integration strategy, mostly the obliging and compromising strategies and, occasionally, the avoidance and domination strategies. The average score of the Patience Scale is considered moderate with a value of 39.15 ± 6.09, and its subdimension scores were determined as 9.88 ± 2.42 for short-term patience, 18.02 ± 2.98 for interpersonal patience, and 11.24 ± 2.35 for long-term patience, respectively. Participants' level of tranquillity is considered moderate, with a score of 3.70 ± 0.70. In addition, their average scores from the scales are shown in Table 1.

Accordingly, executive nurses' long-term, short-term, and total patience levels were significantly lower in case of conflict with the nurses (*p*=0.002, *p*=0.007, and *p*=0.001). Their interpersonal patience levels were also substantially lower in case of conflict with other employees (*p*=0.037). There was no significant difference in the Patience Scale according to gender, marital status, educational status, and being patient and peaceful. No significant difference was found in the Tranquillity Scale by the independent variables. (Table 2).

According to the analysis in Table 3, there was a significant difference in terms of using the strategies of integration and obliging as conflict resolution methods as to the educational status of the executive nurses (*p*=0.040, *p*=0.049). While this difference was observed between postgraduates and high school and undergraduates regarding the integration strategy, it was only seen between postgraduates and undergraduates in the obliging strategy. The table also reveals that postgraduates use the integration and obliging strategies less often. There was no significant difference between conflict resolution strategies by gender, marital status, conflict with nurses and other employees, and state of being patient and peaceful variables. (Table 3).

The corelation analaysis revealed that a weak negative correlation was determined between the variable of working time in the institution and both compromising (*r* = −0.34) and obliging (*r* = −0.37). There was a weak negative (*r* = −0.37) correlation between the working time as a manager variable and the obliging strategy. A weak positive (*r* = 0.35) correlation was determined between short-term patience and the level of tranquillity.

Then again, a moderate positive correlation was found between the strategies of integration and compromising (*r* = 0.66), a strong correlation with obliging (*r* = 0.77), and a weak positive correlation with the level of tranquillity (*r* = 0.31). The correlation analysis is shown in Table 4.

## 4. Discussion

This study, which investigated the strategies of nurses working in managerial positions in two different hospitals in conflict management and their levels of patience and tranquillity, revealed that 51.2% of the participants had conflict with their colleagues and 46.3% with other employees. In similar studies, 61.4% of the nurses in charge of the service stated that they had conflicts with the other nurses [11], and conflict was experienced by all critical care nurses, with 42.5% of the studied nurses having had moderate conflict [44]. This can be explained by the fact that different professional members work together in the health sector, working conditions are complex, and the workload is high. As per the study, the participants mostly used the “integration” strategy in conflict management, the second most “obliging,” and the least “dominating.” Similar to this study, in other relevant studies, it was stated that the strategies of integration and compromising [11, 12, 25, 45–48] were used the most, and the domination [12, 25] strategy was used the least. In addition, some studies indicate the least used strategy was avoidance [11, 45, 46]. The fact that the nursing students mainly used the compromising strategy and least the domination strategy shows similarity with our results [26]. According to the literature, strategic solutions can be solved by integrating and obliging [1]. Consistent with this information, executive nurses use the strategies desired in conflict resolution more. Although administrative nurses and nursing students use the compromising strategy more often in conflict resolution, the data show that conflict continues at high rates in hospitals. Therefore, it is crucial to attempt to solve the individual and organisational factors that are considered to be the cause of this situation. In a study, it was shown that by using tele-nursing application, it can improve the delivery of health services by increasing access to specialised services where mutual communication and interaction are well established [49]. Similarly, tele-nursing application can be tried in conflict resolution.

According to our study, postgraduate executive nurses used “integration” and “obliging” strategies less than high school and undergraduate nurses. While it was similar in one study that undergraduates used the compromising strategy less than other strategies [46], the educational status variable did not make a significant difference in conflict resolution strategies in other studies [11, 45, 47]. This difference may be due to the different grouping of the educational status variable. The fact that postgraduates are positioned in senior management is not unexpected. On the other hand, executives are more likely to resolve strategic conflicts involving differences in planning and objectives, and the literature suggests using the strategies of integration and obliging in strategic conflict resolution. Integration and compromise styles allow all employees to adopt the organisation's goals and objectives. So, the reason for the decrease in integration and generous strategies, which were widely used by students and postgraduates, should be investigated in other studies.

As the duration of work in the profession and the hospital increases, the usage of the strategies of compromising and obliging decreases. Similarly, one study found that the compromising strategy decreased as the experience and seniority increased, which supports our study results [46]. This study has also revealed that the compromising strategy decreased as the working time as a manager increased. According to another study, while the generous strategy was used more in those who worked as a manager for 20 years or more, avoidance was used more in those who worked for 6–10 years [45]. Working as a long-term manager can increase organisational commitment and the use of a compromising strategy. The difference in this study may be that nurses have less managerial experience. Besides, there are studies in which these variables do not make a difference [47].

In this study, the age average was 37, and no significant relationship was found between age and conflict resolution strategies. There are other supporting studies with the same results [47]. However, as a result of this study, the compromising strategy was used significantly more by employees over 45 [45]. The majority of the young population in this study, which showed a positive correlation between age and experience, may explain the difference from the other research.

In the study, similar to the literature, the gender [27] and marital status [45] variables did not make a significant difference in conflict management strategies. Unlike this study, there are studies where males prefer to use the generous [46] or avoidance [47] strategies more, while females prefer to use the integration strategy more [47].

92.7% of the participants think that they are patient. When we look at the scale scores, their patience levels are above average in total and subdimensions, similar to a study [33]. Also, it was observed that although executive nurses' long-term, short-term, and total patience levels decreased during a conflict with the nurses, their interpersonal patience levels did not change. These results suggest that executive nurses maintain communication with nurses by looking at events situationally to ensure the continuity of their work. Their interpersonal patience levels decrease during a conflict with other employees. This may suggest that they individualise the events more. The literature shows a positive relationship between patience and decision-making skills [50]. Therefore, the decrease in the interpersonal patience levels of executive nurses may affect their problem-solving skills and create new clash environments. In this case, it may lead to communication loss with other employees. The study found no relationship between patience level and conflict resolution. In this case, hypothesis 1 is rejected.

70.7% of the participants in the study stated that they were peaceful, and their tranquillity levels were found to be moderate according to the scale. Also, there was a positive correlation between their tranquillity and short-term patience levels. This finding supports hypothesis 3. During a conflict with nurses whose short-term patience levels are low, there is expected to be a decrease in the tranquillity levels of executive nurses. The fact that their tranquillity level could have been higher in this study may be the fact that they always use the integration strategy. Considering this study, a positive correlation was found between the integration strategy and the level of tranquillity. This relationship supports hypothesis 2.

### 4.1. Limitations and Strengths

Including only public institutions in the study and the small number of managers may be a limitation. However, it is a strength that the issues of patience and peace of mind are addressed together. What makes this work powerful is that no studies have been found in the healthcare field in which conflict management strategies are being studied together with tranquillity and patience. In addition, the fact that studies on patience and tranquillity in nurses are limited in general makes this study vital.

## 5. Conclusion and Recommendations

Accordingly, the participants used all conflict management strategies, mostly “integration” and frequently “obliging” and “compromising.” Also, their educational status was influential in their conflict management strategy preferences. In addition, the patience and tranquillity levels of the participants were moderate. While their long- and short-term patience levels decrease in case of a conflict with nurses, their interpersonal patience levels decrease in a conflict with other employees in the hospital. This study has also revealed that the level of tranquillity increases as the integration strategy is used in conflict resolution. On the other hand, no relationship was found between patience and conflict management strategies. This finding indicates that executive nurses maintain professionalism and exhibit the same attitude in the face of events.

Due to its nature, health services have a complex structure, and it is an area where different disciplines work together, so conflicts are inevitable. They need to be managed in a way that will result in a positive outcome as it will affect cooperation and quality of health service delivery. For tranquillity, activities such as in-service training are recommended to increase the use of “integration” by all managers. It is crucial to draw attention to this issue, especially in the postgraduate education process. A unit can be established within the organisation for staff only, where nurses can receive support when they feel that their level of patience and peace of mind has decreased. It is recommended that similar issues be studied with nurses and other health professionals in future studies. In addition, qualitative research methods can be used to examine the issues in depth.

## Figures and Tables

**Table 1:** Participants' Patience Scale, Rahim Organisational Conflict Inventory Scale, and Tranquillity Scale scores.

Scales	*n*	Minimum	Maximum	χ	Sd.
Patience Scale					
İnterpersonal patience	41	11	21	18.02	2.98
Long-term patience	41	5	15	11.24	2.35
Short-term patience	41	4	15	9.88	2.42
Total patience	41	27	47	39.15	6.09
Rahim Organizational Conflict Inventory (ROCI II)					
İntegration	41	19	30	4.40	0.52
Compromising	41	8	25	3.61	0.79
Dominating	41	6	25	2.56	0.83
Avoiding	41	6	30	2.93	0.82
Obliging	41	18	30	4.04	0.55
Tranquillity Scale	40	16	40	3.70	0.70

**Table 2:** The analysis of the Patience Scale and the Tranquillity Scale with certain variables.

Independent variables	Long-term patience		Short-term patience		İnterpersonal patience		Total patience		Tranquillity Scale	
	Mean ± sd	*t*/*F*	Mean ± sd	*t*/*F*	Mean rank	U-Z/KWX^2^	Mean ± sd	*t*/*F*	Mean ± sd	*t*/*F*
		*p*		*p*		*p*		*p*		*p*
Gender										
Woman	11.24 ± 2.39	*t* = −0.01	10.11 ± 2.27	*t* = 1.91	21.70	*U* = 48.00	39.51 ± 6.05	*t* = 1.18	3.76 ± 0.66	*t* = 1.75
Men	11.25 ± 2.21	*p*=0.996	7.75 ± 3.09	*p*=0.063	14.50	*Z* = −1.16	35.75 ± 6.13	*p*=0.245	3.13 ± 0.97	*p*=0.088
						*p*=0.274				
Marital status										
Married	11.16 ± 2.37	*t* = −0.83	9.79 ± 2.41	*t* = −0.83	21.21	*U* = 49.00	39.00 ± 6.22	*t* = −0.54	3.68 ± 0.73	*t* = −0.45
Single	12.33 ± 2.08	*p*=0.412	11.00 ± 2.64	*p*=0.411	18.33	*Z* = −0.40	41.00 ± 4.35	*p*=0.590	3.88 ± 0.33	*p*=0.655
						*p*=0.723				
Educational status										
High school	13.50 ± 2.12	*F* = 1.07	9.00 ± 5.65	*F* = 0.19	29.00	KWX^2^ = 1.20	42.00 ± 9.89	*F* = 0.21	3.69 ± 1.33	*F* = 0.46
Associate's degree	10.63 ± 1.50	*p*=0.371	10.38 ± 2.61	*p*=0.898	20.69	*p*=0.752	39.13 ± 4.25	*p*=0.889	3.41 ± 0.69	*p*=0.712
Bachelor's degree	11.11 ± 2.63		9.81 ± 2.54		20.17		38.78 ± 6.81		3.75 ± 0.71	
Postgraduates	12.25 ± 0.95		9.75 ± 2.50		23.25		40.25 ± 2.87		3.81 ± 0.60	
Condition of a conflict with nurses										
Yes	10.19 ± 2.18	*t* = −3.27	8.90 ± 2.54	*t* = −2.86	18.05	*U* = 148.00	36.29 ± 5.65	*t* = −3.48	3.58 ± 0.72	*t* = −1.13
No	12.35 ± 2.03	*p*=0.002	10.90 ± 1.83	*p*=0.007	24.10	*Z* = −1.64	42.15 ± 5.08	*p*=0.001	3.83 ± 0.68	*p*=0.266
						*p*=0.101				
Condition of a conflict with other employees										
Yes	11.21 ± 1.98	*t* = −0.08	9.37 ± 2.52	*t* = −1.26	16.87	*U* = 130.50	37.89 ± 5.02	*t* = −1.23	3.51 ± 0.69	*t* = −1.65
No	11.27 ± 2.67	*p*=0.934	10.32 ± 2.29	*p*=0.214	24.57	*Z* = −2.08	40.23 ± 6.81	*p*=0.226	3.87 ± 0.68	*p*=0.106
						*p*=0.037				
Are you patient										
Yes	11.18 ± 2.35	*t* = −0.57	9.95 ± 2.49	*t* = 0.64	21.03	*U* = 56.00	39.16 ± 6.32	*t* = 0.04	3.70 ± 0.72	*t* = 0.18
No	12.00 ± 2.64	*p*=0.570	9.00 ± 1.00	*p*=0.521	20.67	*Z* = −0.05	39.00 ± 1.00	*p*=0.966	3.63 ± 0.66	*p*=0.857
						*p*=0.981				
Are you peaceful										
Yes	11.48 ± 2.21	*t* = 1.01	10.24 ± 2.48	*t* = 1.51	23.26	*U* = 108.50	40.03 ± 6.13	*t* = 1.47	3.75 ± 0.72	*t* = 0.66
No	10.67 ± 2.67	*p*=0.318	9.00 ± 2.08	*p*=0.137	15.54	*Z* = −1.90	37.00 ± 5.64	*p*=0.149	3.58 ± 0.68	*p*=0.513
						*p*=0.060				

sd: standard deviation, KW: Kruskal–Wallis, U: Mann–Whitney U.

**Table 3:** The analysis of conflict resolution strategies with certain variables.

Independent variables	Compromising		Dominating		İntegration		Avoiding		Obliging	
	Mean ± sd	*t*/*F*	Mean ± sd	*t*/*F*	Mean rank	U-Z/KWX^2^	Mean ± sd	*t*/*F*	Mean ± sd	*t*/*F*
		*p*		*p*		*p*		*p*		*p*
Gender										
Woman	3.64 ± 0.81	*t* = 0.69	2.53 ± 0.86	*t* = −0.72	21.41	*U* = 59.00	2.95 ± 0.86	*t* = 0.55	4.05 ± 0.56	*t* = 0.15
Men	3.35 ± 0.72	*p*=0.490	2.85 ± 0.44	*p*=0.472	17.25	*Z* = −0.666	2.71 ± 0.44	*p*=0.584	4.00 ± 0.53	*p*=0.879
						*p*=0.505				
Marital status										
Married	3.62 ± 0.77	*t* = 0.03	2.55 ± 0.85	*t* = −0.22	21.11	*U* = 53.00	2.93 ± 0.81	*t* = −0.03	4.04 ± 0.55	*t* = 0.13
Single	3.60 ± 1.25	*p*=0.974	2.67 ± 0.70	*p*=0.823	19.67	*Z* = −0.202	2.94 ± 1.18	*p*=0.970	4.00 ± 0.60	*p*=0.896
						*p*=0.840				
Educational status										
High school	3.60 ± 1.13	*F* = 1.21	1.90 ± 0.42	*F* = 0.45	28.25	KWX^2^ = 8.297	3.00 ± 0.24	*F* = 0.32	3.83 ± 0.71	*F* = 2.87
Associate's degree	3.53 ± 0.46	*p*=0.318	2.53 ± 0.64	*p*=0.719	17.19	*p*=0.040	2.79 ± 0.81	*p*=0.811	4.00 ± 0.49	*p*=0.049
Bachelor's degree	3.74 ± 0.85		2.61 ± 0.93		23.63		3.01 ± 0.88		4.17 ± 0.53	
Postgraduates	2.95 ± 0.62		2.65 ± 0.59		7.25		2.63 ± 0.84		3.38 ± 0.37	
Condition of a conflict with nurses										
Yes	3.56 ± 0.67	*t* = −0.43	2.69 ± 0.83	*t* = 0.98	19.05	*U* = 169.0	3.02 ± 0.66	*t* = 0.76	3.99 ± 0.53	*t* = −0.57
No	3.67 ± 0.92	*p*=0.669	2.43 ± 0.83	*p*=0.332	23.05	*Z* = −1.081	2.82 ± 0.97	*p*=0.448	4.09 ± 0.58	*p*=0.570
						*p*=0.280				
Condition of a conflict with other employees										
Yes	3.49 ± 0.71	*t* = −0.89	2.57 ± 0.82	*t* = 0.05	19.50	*U* = 180.5	2.92 ± 0.81	*t* = −0.04	3.97 ± 0.54	*t* = −0.71
						*Z* = −0.753				
No	3.72 ± 0.86	*p*=0.376	2.55 ± 0.86	*p*=0.958	22.30	*p*=0.451	2.93 ± 0.86	*p*=0.967	4.10 ± 0.56	*p*=0.477
Are you patient										
Yes	3.66 ± 0.79	*t* = 1.25	2.50 ± 0.83	*t* = −1.71	21.74	*U* = 29.00	2.95 ± 0.85	*t* = 0.56	4.06 ± 0.09	*t* = 0.85
No	3.07 ± 0.61	*p*=0.219	3.33 ± 0.11	*p*=0.095	11.67	*Z* = −1.417	2.67 ± 0.50	*p*=0.578	3.78 ± 0.43	*p*=0.398
						*p*=0.156				
Are you peaceful										
Yes	3.63 ± 0.88	*t* = 0.16	2.63 ± 0.87	*t* = 0.79	22.16	*U* = 140.5	2.95 ± 0.88	*t* = 0.32	4.10 ± 0.61	*t* = 1.138
No	3.58 ± 0.58	*p*=0.873	2.40 ± 0.74	*p*=0.433	18.21	*Z* = −0.971	2.86 ± 0.71	*p*=0.748	3.89 ± 0.34	*p*=0.262
						*p*=0.332				

sd: standard deviation, KW: Kruskal–Wallis, U: Mann–Whitney U.

**Table 4:** The correlation between independent variables, the Patience Scale, the Tranquillity Scale, and the Rahim Organisational Conflict Inventory Scale.

Variables		Working time in the profession	Working time in the current institution	Active time as a manager	İnterpersonal patience	Long-term patience	Short-term patience	Total patience	İntegration	Compromising	Dominating	Avoiding	Obliging	Tranquillity level
Age	*r*	0.77^*∗∗∗*^	0.69^*∗∗∗*^	0.49^*∗∗*^	0.01	−0.04	−0.12	−0.06	−0.21	−0.23	−0.05	−0.08	−0.24	−0.03
Working time in the profession	*r*	1.00	0.88^*∗∗∗*^	0.67^*∗∗∗*^	0.01	−0.02	−0.09	−0.04	−0.23	−0.34^*∗*^	−0.17	−0.17	−0.37^*∗*^	0.04
Working time in the current institution	*r*		1.00	0.73^*∗∗∗*^	0.1	0.05	−0.06	0.06	−0.22	−0.34^*∗*^	−0.13	−0.20	−0.37^*∗*^	0.02
Working time as a manager	*r*			1.00	0.07	0.14	0.05	0.11	−0.24	−0.26	−0.16	−0.17	−0.34^*∗*^	0.08
İnterpersonal patience	*r*				1.00	0.52^*∗∗∗*^	0.47^*∗∗*^	0.88^*∗∗∗*^	0.13	0.07	0.15	0.17	0.22	−0.07
Long-term patience	*r*					1.00	0.22	0.73^*∗∗∗*^	−0.01	−0.06	0.16	0.02	0.09	0.11
Short-term patience	*r*						1.00	0.71^*∗∗∗*^	0.02	0.08	0.12	0.08	0.06	0.35^*∗*^
Total patience	*r*							1.00	0.07	0.04	0.19	0.12	0.17	0.14
İntegration	*r*								1.00	0.66^*∗∗∗*^	0.10	0.27	0.77^*∗∗∗*^	0.31^*∗*^
Compromising	*r*									1.00	0.26	0.65^*∗∗∗*^	0.70^*∗∗∗*^	0.16
Dominating	*r*										1.00	0.54^*∗∗∗*^	0.40^*∗∗*^	0.11
Avoiding	*r*											1.00	0.50^*∗∗*^	−0.09
Obliging	*r*												1.00	0.11

^*∗*^ *p* < 0.05, ^*∗∗*^*p* < 0.01, ^*∗∗∗*^*p* < 0.001.

## Data Availability

The data used to support the findings of this study are available from the corresponding author upon reasonable request.
